# Molecular contacts in self-assembling clusters of membrane proteins

**DOI:** 10.1073/pnas.2507112122

**Published:** 2025-06-23

**Authors:** Venkata Shiva Mandala, Ziao Fu, Roderick MacKinnon

**Affiliations:** ^a^Laboratory of Molecular Neurobiology and Biophysics, The Rockefeller University, New York, NY 10065; ^b^HHMI, The Rockefeller University, New York, NY 10065

**Keywords:** HOTS, membrane proteins, clustering, cryo-EM

## Abstract

Motivated by recent data pointing to the existence of homo-oligomeric assemblies of membrane proteins called higher-order transient structures, and their apparent role in connecting components of membrane signal pathways, we examine here by cryoelectron microscopy some of the protein–protein interactions that occur in cluster formation. Metabotropic glutamate receptors and HCN ion channels inside clusters contact their neighbors through structured extracellular and intracellular domains, respectively. Other ion channels, including Kv2.1 and Slo1, appear to form clusters through prominent intrinsically disordered sequences in the cytoplasm. These distinct modes of interaction are associated with clusters exhibiting varying degrees of compactness and order. We conclude that nature utilizes a variety of ways to form connections between membrane proteins in self-assembled clusters.

## Significance

Many proteins function not just in isolation but also by interacting with other proteins as part of signaling pathways in living cells. Much is already known about the structures of different proteins and what role they play in organisms. But molecular details of how different proteins interact with each other to form larger assemblies are not well understood. Here, we study how several different membrane proteins self-interact to form clusters. We find that proteins contact each other in a variety of ways—involving both ordered and disordered regions—to form these self-clusters, highlighting the diversity of chemical interactions nature uses in the formation of protein assemblies in cell membranes.

We recently published two papers showing that many membrane proteins appear to self-assemble spontaneously to form homo-oligomers in the cell membrane (1, 2). These oligomers are not required for the basic catalytic function of the proteins involved. For example, most class A G-protein coupled receptors (GPCRs) are activatable as monomers by their cognate ligand and split G protein trimers into active G protein substituents (3, 4). And yet we observed that several GPCRs assemble to form oligomers such that, on average, they are present as small clusters of self (homo-oligomers) in the cell membrane rather than being randomly distributed (1). For the muscarinic type 2 GPCR (M2R), our data support the proposal that the clusters are required to produce enough Gβγ locally (surrounding a cluster) to activate nearby G protein–gated K^+^ (GIRK) channels (2, 5, 6). The GIRK channels themselves also form small clusters even though single channels function perfectly normally (1, 2). We think that GIRK channels, by forming clusters, have a higher of chance of being nearby M2R clusters because weak interactions between GIRK and M2R are enhanced by the multivalence of clusters (2). Thus, clusters can enhance signaling from M2R to the GIRK channel, which is important for controlling heart rate. We propose this is why clusters are formed. Next, we wish to understand how they are formed.

The membrane protein clusters, also referred to here as oligomers, exhibit the following properties. First, they are specific, meaning each protein forms clusters of identical proteins. Second, the free energy changes associated with the transfer of an isolated protein unit to a cluster are small, around a few k_B_T, causing the clusters to be readily reversible. Weak interactions account for a characteristic cluster size distribution and for the transience of this kind of cluster. We call these higher-order transient structures (HOTS) (1).

The first property of HOTS, specificity, is most easily explained if the proteins that form HOTS have regions, either specific amino acids or complementary shapes that allow them to interact with themselves, although other explanations are possible. In our initial studies, we could tell that proteins in HOTS can be close enough to touch each other (1), but we could not look at the proteins themselves in detail to study how they interact. In the present paper, we take a closer look. Using cryoelectron microscopy (cryo-EM) we examine four different proteins in membranes: a class C GPCR and three ion channels. Our goal here is to ask whether clustering is mediated by direct protein–protein interactions and whether there are specific rules governing how such interactions occur?

## Results

### Molecular Contacts Are the Same in HOTS Clusters and Bulk Phase Clusters.

HOTS refer to the clusters resulting from self-assembly of weakly interacting proteins in the subcritical regime, that is, when the concentration of the membrane protein is not too high. The HOTS cluster size distribution contains monomers, dimers, trimers, etc., in progressively decreasing concentrations. As we have shown (1, 2), when the total concentration of a membrane protein exceeds its critical concentration, then larger, bulk phase clusters can appear in equilibrium with the distribution of HOTS. This phenomenon occurs because at the critical concentration, the chemical potential of a protein in the HOTS distribution equals that of a protein in the bulk phase. In the absence of other factors, interactions between individual proteins in bulk phase clusters and in HOTS should in principle be the same, because the very same equilibrium process drives the formation of HOTS and bulk phase clusters (1, 7–9). In the present study, we express or reconstitute proteins to levels clearly exceeding their critical concentration, so most of the proteins we will look at are in the form of bulk phase clusters. We emphasize that neighboring protein contacts that form bulk phases should be the same as those interactions that form HOTS—in other words, the molecular interactions between proteins in the interior of a cluster should be independent of the size of the cluster.

### mGluR7 Interacts via Its Extracellular Domain (ECD) in Both Cell-Derived and Synthetic Membranes.

We look first at the metabotropic glutamate receptor mGluR7, a class C GPCR important for synaptic transmission and neuronal development in the central nervous system (10). mGluR7 forms a functional dimer with extracellular domains (ECDs) responsible for ligand binding and dimerization (the ECDs in the dimer are disulfide bridged), transmembrane domains (TMDs) that mediate coupling to G-proteins, and short intracellular domains (ICDs) that are largely unstructured (11). Dimers of mGluR7 have been shown to form immobile clusters at presynaptic sites in neurons and the ECDs have been implicated in this behavior (12). Replacing the ECD of mGlur7 with the corresponding domain from mGluR1 or mGluR2, which are less clustered compared to mGluR7, increases both the diffusion coefficient of mGluR7 dimers as well as the fraction of dimers that are mobile (12). Thus, the ECD of mGluR7 has been shown to be important for cluster formation in cells. But molecular details of mGluR7 assembly are not known.

**Fig. 1.**
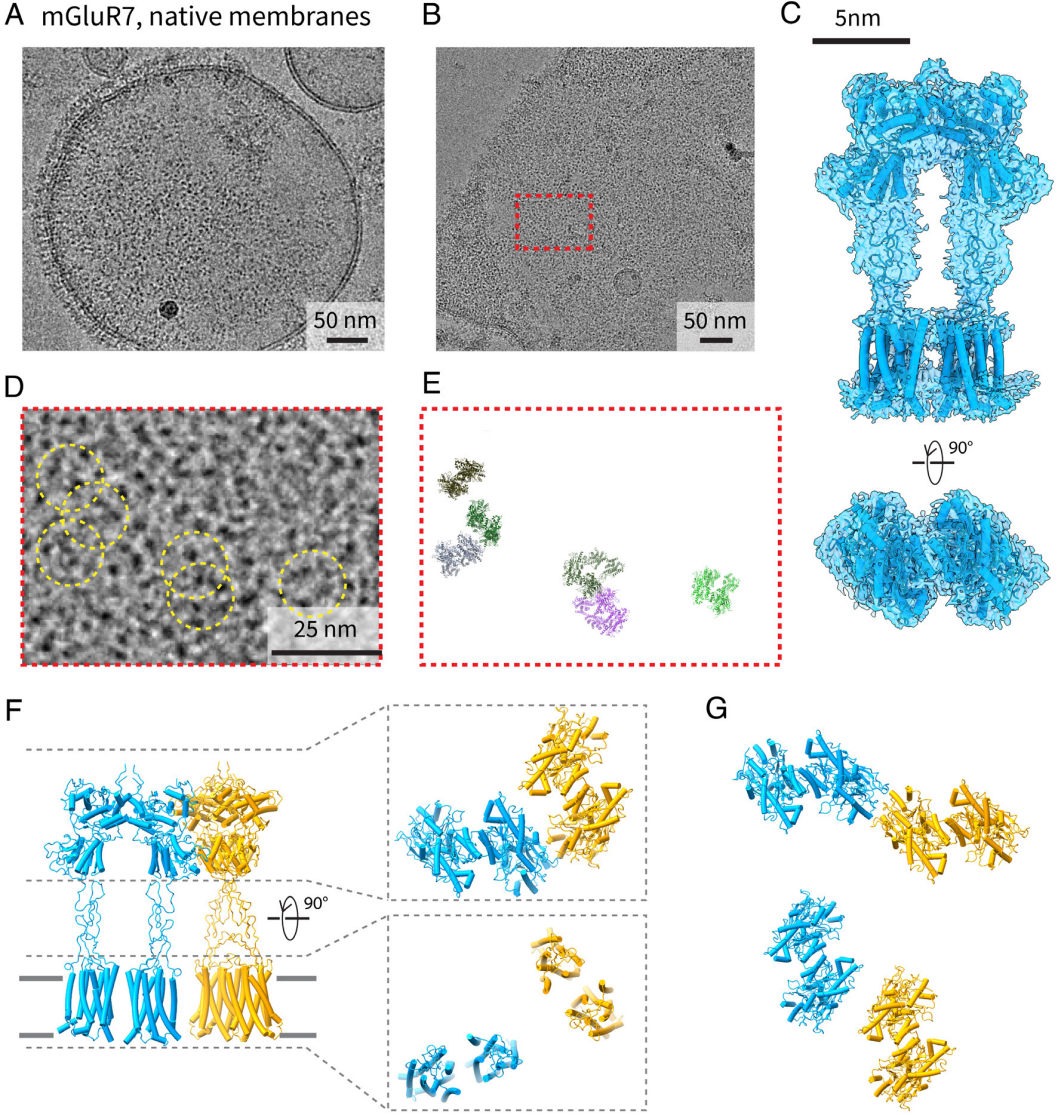
Clusters of mGluR7 in NMVs. (*A*) Cryo-EM micrograph of NMVs containing mGluR7s. The receptors form a large bulk phase cluster that is absent in the *Upper Right* quadrant. (*B*) Another micrograph showing a large mGluR7 cluster in a vesicle. (*C*) Cryo-EM reconstruction of the mGluR7 dimer in NMVs. The density map is shown in blue as a translucent surface and the structure of mGluR7 is shown in cartoon representation in the same color. (*D*) Zoomed-in region from panel *B* (red box) showing individual particles (marked by yellow circles) that were used in the cryo-EM reconstruction in panel *C*. (*E*) Zoomed-in region from panel *B* (red box) showing the orientation and locations of individual particles that were included in the density map. (*F*) Two dimers that are close enough to interact via their ECDs. Each dimer is shown in a different color in cartoon representation. (*G*) Other interacting dimer pairs showing that many interaction modes are possible with the ECDs.

To address the mechanisms of assembly, we purified native membrane vesicles (NMVs) derived from cells expressing excess mGluR7 (Fig. 1). NMVs were generated as described before using N-ethylmaleimide (NEM)/Ca^2+^ and those containing mGluR7 were enriched using affinity chromatography (*SI Appendix*, Fig. S1*C*) (13–15). mGluR7 clearly forms bulk phase clusters in the NMVs at these high concentrations (Fig. 1 *A* and *B*). Selected proteins within the clusters permit calculation of a cryo-EM density map of an mGluR7 dimer (Fig. 1*C* and *SI Appendix*, Fig. S2*A*). Using the single molecule translations and angles from image analysis and map refinement to position dimers in the raw images (Fig. 1 *D* and *E*), neighboring pairs of mGluR dimers can be seen to contact each other mainly through the large extracellular ligand binding domains (Fig. 1*F*). The contact sites appear multiple, instead of being dominated by a particular pair of surfaces of the ligand binding domains (Fig. 1*G*).

**Fig. 2.**
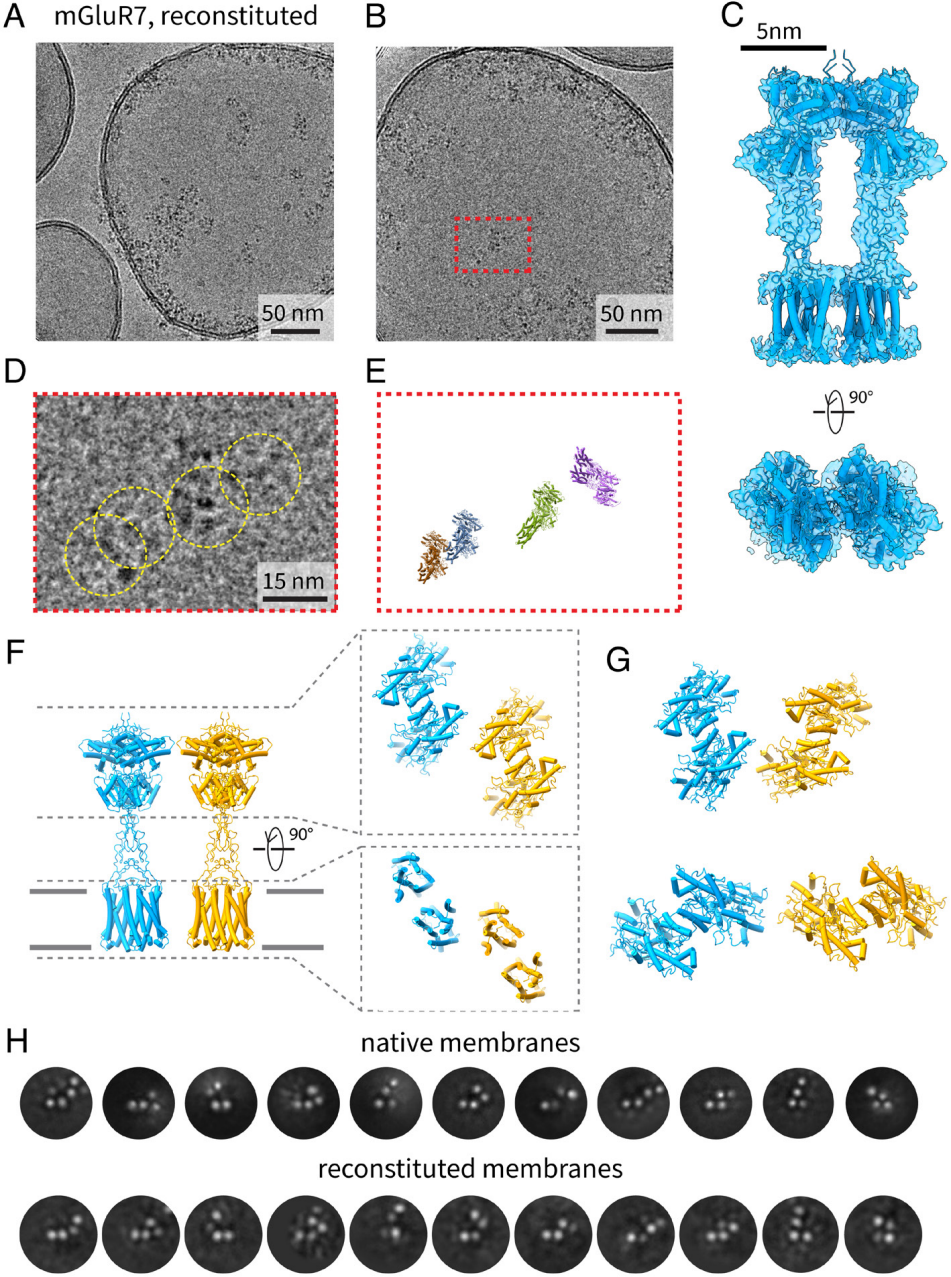
Clusters of mGluR7 reconstituted into synthetic (POPC) membranes. (*A*) Cryo-EM micrograph of POPC vesicles containing mGluR7s. The receptors insert in both orientations but still form clusters. (*B*) Another image with mGluR7 clusters in a synthetic vesicle. (*C*) Cryo-EM reconstruction of the mGluR7 dimer in POPC vesicles. The density map is shown in blue as a translucent surface and the structure of mGluR7 is shown in cartoon representation in the same color. (*D*) Zoomed-in region from panel *B* (red box) showing individual particles (marked by yellow circles) that were used in the cryo-EM reconstruction in panel *C*. (*E*) Zoomed-in region from panel *B* (red box) showing the orientation and locations of individual particles that were included in the density map. (*F*) Two dimers that are close enough to interact via their ECDs. Each dimer is shown in a different color in cartoon representation. (*G*) Other interacting dimer pairs showing that many interaction modes are possible with the ECDs. (*H*) Two-dimensional (2D) class averages showing similar pairs of interacting dimers in NMVs (*Top* row) and in synthetic (POPC) vesicles (*Bottom* row).

Micrographs with side views of the mGluR7 bulk phase clusters (Fig. 1*A*) show strong protein density on the intracellular side of the NMV that does not appear in the final cryo-EM reconstruction of an mGluR7 dimer. While mGluR7 dimers contain only a small number of amino acids on the intracellular side, it is possible that other proteins from the cytoplasm—purified along with the NMVs—could mediate cluster formation. To test whether mGluR7 requires other proteins or native cellular lipids to form clusters, dimers were purified to homogeneity in detergent (*SI Appendix*, Fig. S1 *A* and *B*) and then reconstituted into synthetic lipid vesicles (Fig. 2 *A* and *B*) composed of a single type of lipid [1-palmitoyl-2-oleoyl-glycero-3-phosphocholine (POPC)].

The clusters of purified mGluR7 dimers in a synthetic lipid membrane (Fig. 2 *A* and *B*) appear much the same as those in NMVs (Fig. 1 *A* and *B*). The primary difference is that in reconstituted vesicles mGluR7 dimers can appear in the membrane in both orientations—inside-out and outside-out—but clusters tend to form between similarly oriented proteins. We calculated a cryo-EM density map for the dimer in synthetic vesicles (Fig. 2*C* and *SI Appendix*, Fig. S2*B*), which allowed us to perform the same reconstruction-based analysis as before (Fig. 2 *D* and *E*). Again, contacts between neighboring mGluR dimers inside bulk phase clusters form predominantly through interfaces formed by the ECDs (Fig. 2 *F* and *G*). Selected two-dimensional class averages of adjacent dimers derived from NMVs (Fig. 2 *H*, *Top* row) and reconstituted vesicles (Fig. 2 *H*, *Bottom* row) emphasize the large number of distinct ways the ECDs can contact each other and the similarity of contacts in both the “native” and reconstituted systems (deduced from the similar dimer pairs observed in the two preparations). We do not know the relative energetic importance of the different contact interfaces. The structures only tell us that multiple contact surfaces are present within clusters.

### HCN1 Channels Form a Regular Lattice in Synthetic Membranes.

We next turned to the tetrameric cation channel HCN1, which plays important roles in neuronal excitability and cardiac and neuronal pacemaker activity (16). HCN channels are also known to be highly localized in various tissues—for instance, HCN1 channels are concentrated at the inner segment of rod photoreceptors and at axon terminals in bipolar cells (17, 18).

**Fig. 3.**
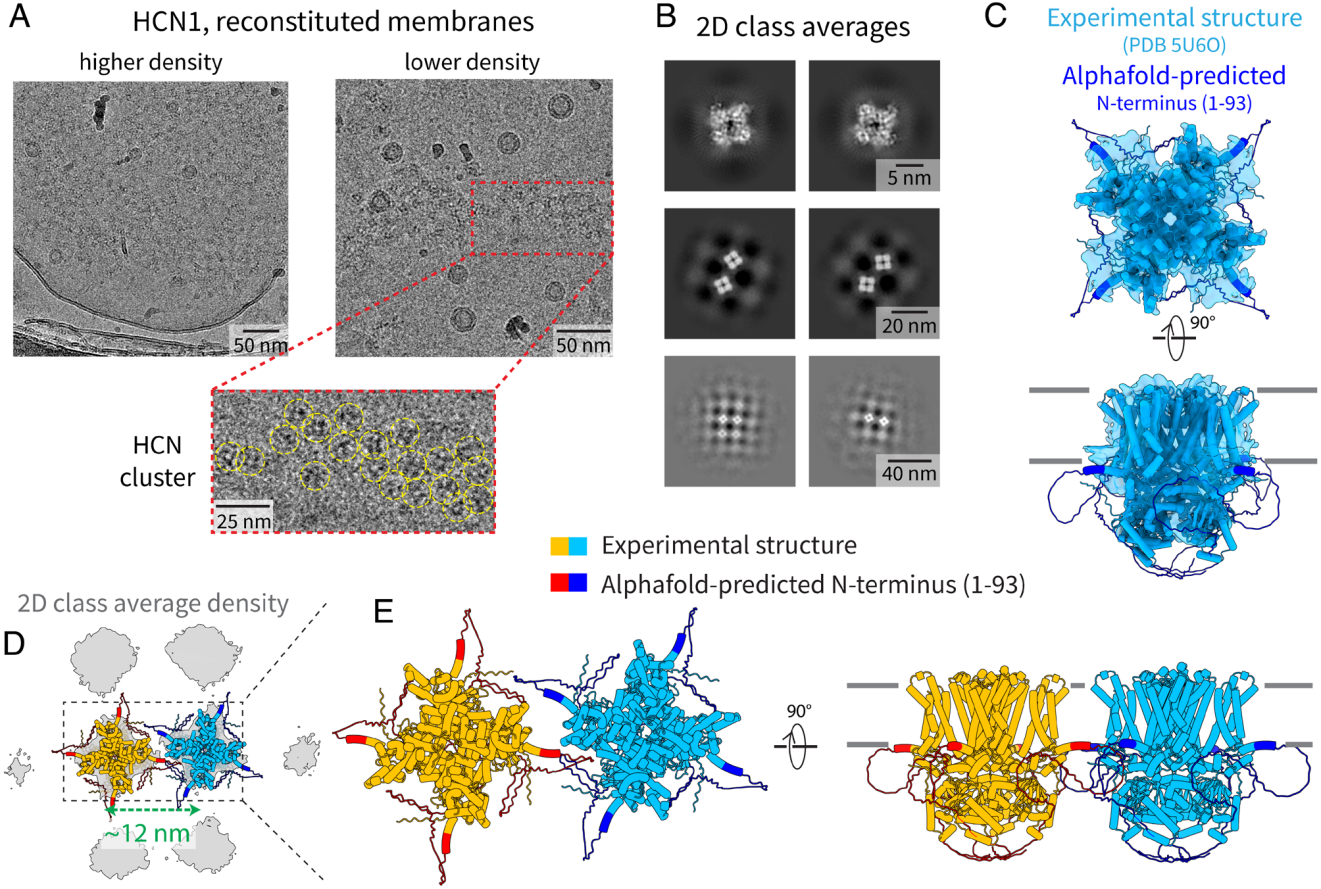
Ordered clusters of HCN1 in POPC membranes. (*A*) Representative cryo-EM images of POPC membranes containing HCN1 channels at higher density (*Left*) or at lower density (*Right*). An expanded view of an HCN1 cluster from the membrane with lower HCN1 density is shown in the red box. Individual HCN1 channels in the cluster are marked by yellow circles. (*B*) Two-dimensional class averages at different length scales, showing individual channels (*Top* row), interacting pairs of channels (*Middle* row), and the structure of the ordered lattice (*Bottom* row). (*C*) Cryo-EM reconstruction of the tetrameric HCN1 channel in POPC vesicles. The density map is shown in blue as a translucent surface and the structure of HCN1 is shown in cartoon representation in the same color. The AlphaFold-predicted N terminus (residues 1 to 93) that is not resolved in the detergent structure is shown in dark blue. (*D*) 2D cryo-EM density of the HCN1 lattice is shown in gray, and the two channels that were placed into the lattice are shown in cartoon representation. (*E*) Structural model of the two interacting HCN1 channels shown in cartoon representation with each in a different color. The detergent structure of HCN1 is shown in orange/light blue and the AlphaFold-predicted N terminus is shown in red/dark blue. The channels are not close enough to interact via their transmembrane or visible cytoplasmic domains but rather via contact sites close to the N terminus of the channel.

To see whether these channels self-assemble, HCN1 was expressed recombinantly, purified to homogeneity in detergent, and reconstituted into lipid vesicles composed of a single lipid (POPC). HCN1 channels form highly ordered, almost quasi-crystalline clusters in these membranes (Fig. 3*A*). Individual channels and channel pairs inside clusters were selected for image processing (Fig. 3*B*). Two-dimensional class averages of tetramer pairs show that a regular lattice results because the fourfold symmetric channel contacts its neighbors mainly through a single dominant surface near the four (identical) corners of the square tetramer (Fig. 3 *B* and *D*).

To study the contacts between channels, we first calculated a cryo-EM map from individual tetramers (Fig. 3*C* and *SI Appendix*, Fig. S2*C*). The known structure of HCN1 in detergent micelles (19) (blue) was docked into the map (Fig. 3*C*), which showed additional density near the N terminus of the channel (AlphaFold prediction (20) shown in dark blue in Fig. 3*C*). The channels can also be mapped back to the lattice (Fig. 3*D*), where it is apparent that the TMDs and ICDs from adjacent tetramers are too far to contact each other. Rather, the extra density near the N terminus likely mediates the contact between channels in the lattice.

Thus, we see that the situation for self-assembled clusters of HCN1 contrasts that for mGluR7. In HCN1, a single surface (replicated four times by the channel’s symmetry) located on the intracellular side of the membrane dominates the interaction between neighbors in the cluster. This surface appears to be fairly ordered. The combination of molecular symmetry and one dominant relatively ordered surface gives rise to the growth of crystal-like clusters. In mGluR7, the contacts occur on the extracellular side. The mGluR7 dimer has twofold symmetry, and the contacts are made through ordered structures on the protein. However, in contrast to HCN1, there appear to be many competing contact surfaces in mGluR7 rather than a dominant favorable surface. Consequently, clusters of mGluR7 are not arrayed with the kind of regularity observed for HCN1.

### Kv2.1 Channels Contact Each Other Through Disordered Regions.

The neuronal voltage-gated potassium channel Kv2.1, like HCN1, is a tetramer with four identical subunits (21). Previous studies have shown that it forms clusters in neurons and that the formation of these clusters in cells is controlled by the extended, disordered C-terminus of this channel (22, 23). Specifically, chimeras of Kv2.1 with other Kv channels that do not cluster in cells (including the closely related Kv2.2) showed that the disordered C-terminus of Kv2.1 is both necessary and sufficient for clustering (22–24). The ability of Kv2.1 to form self-clusters can thus be transferred onto other proteins by bestowing the C-terminus on them.

**Fig. 4.**
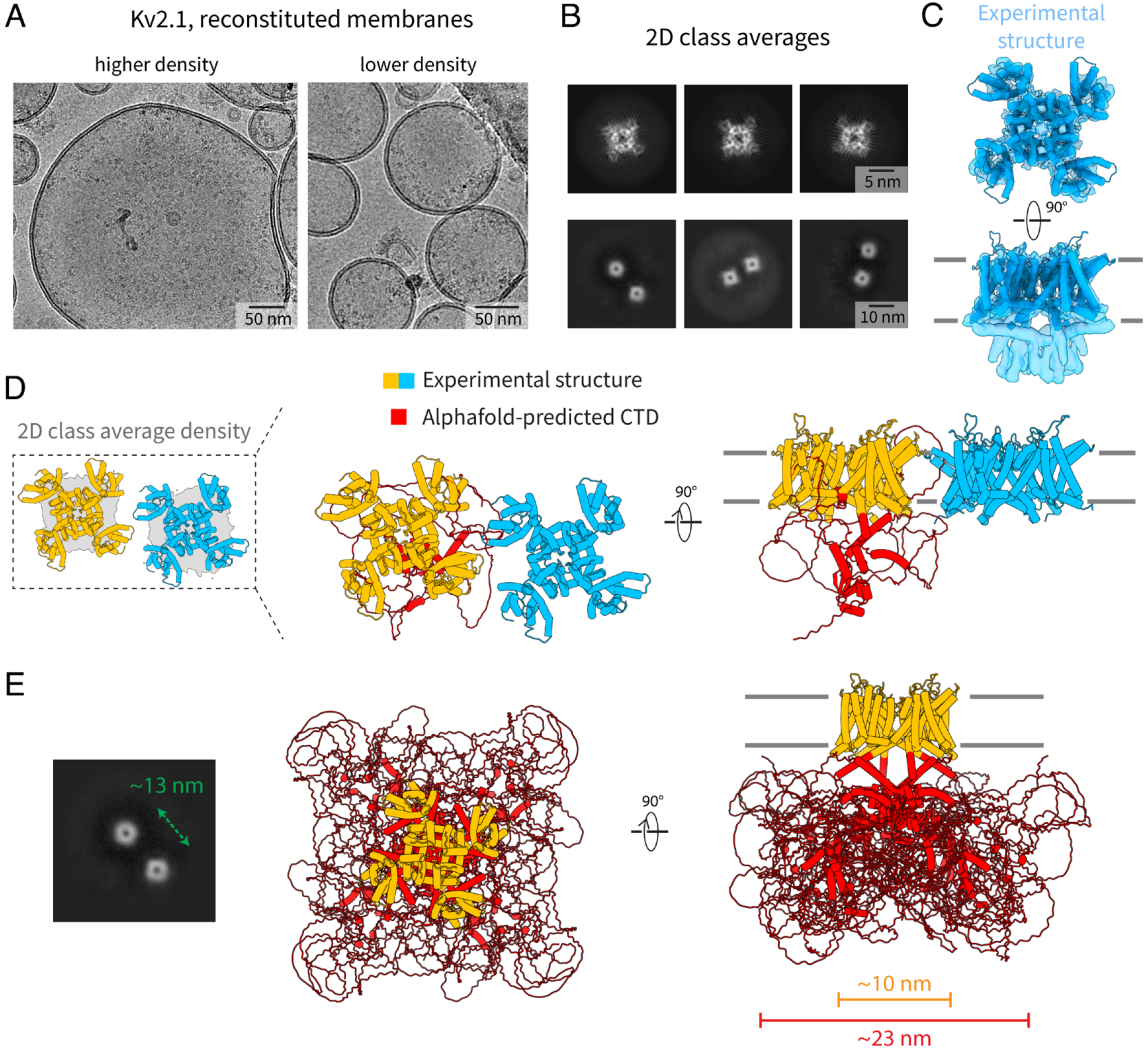
Clusters of Kv2.1 channels in POPC membranes. (*A*) Representative cryo-EM images of POPC membranes containing Kv2.1 channels at higher density (*Left*) or at lower density (*Right*). (*B*) Two-dimensional class averages showing individual channels (*Top* row) and interacting pairs of channels (*Bottom* row). (*C*) Cryo-EM reconstruction of the tetrameric Kv2.1 channel. The density map is shown in blue as a translucent surface and the structure of Kv2.1 in membranes is shown in cartoon representation in the same color. (*D*) 2D class average of the pair of channels that are closest to each other is shown in gray, with the two molecules that were placed into the map shown in cartoon representation. The expanded view shows a structural model of the two interacting Kv2.1 channels shown in cartoon representation. The structure of Kv2.1 in membranes is shown in orange/light blue and the AlphaFold-predicted cytoplasmic domain and C-terminus are shown in red for one subunit in the orange channel. (*E*) 2D class average (*Left*, reproduced from panel *B*) of a pair of channels that are further apart, showing that the ordered domains are too far apart to touch each other. A structural model of a single Kv2.1 tetramer (*Right*) showing the ordered transmembrane structure in orange, and the AlphaFold-predicted cytoplasmic domain and C-terminus in red, to show the expanded “footprint” of the channel. To emphasize the flexible nature of the C-terminal loops, four AlphaFold-predicted models are overlaid.

We recently showed that Kv2.1 forms HOTS clusters as well as bulk phase clusters in reconstituted lipid vesicles (1). Here, we take a closer look at how these channels self-interact, by purifying Kv2.1 channels and reconstituting them into synthetic membrane vesicles composed of POPC (Fig. 4*A*). Isolated channels show well-resolved two-dimensional class averages when viewed from the top or bottom (Fig. 4*B*). Only the structure of the ordered TMD can be resolved (Fig. 4 *C* and *D* and *SI Appendix*, Fig. S2*D*) (25). Part of the cytoplasmic domain is partially visible in the cryo-EM map (Fig. 4*C*), but the extended C-terminus, which comprises about two-thirds of amino acids in the protein, is not visible (Fig. 4 *D* and *E*).

Meanwhile, two-dimensional class averages of tetramer pairs (Fig. 4*B*) are poorly resolved, with both the distance and relative orientations between adjacent Kv2.1 channels inside clusters being highly variable. Some pairs are close enough to perhaps make direct contact through helices near the membrane surfaces (Fig. 4*D*), but others are too far away (Fig. 4*E*). This apparent distal interaction is thus best explained by the large disordered intracellular C-terminus of Kv2.1 (Fig. 4 *D* and *E*), which, when mutated, has been shown to affect its ability to form clusters (22, 23). It is apparent that the footprint of the channel (Fig. 4*E*) is much larger when considering the unresolved disordered regions (~23 nm) compared to just the ordered parts of the protein (~10 nm).

In membrane biology, intrinsically disordered regions (IDRs) are thought to mediate interactions with cytoskeletal components and other scaffold proteins (26). In soluble proteins, they are associated with aggregation (e.g., biomolecular condensates, a form of bulk phase cluster formation) (27–30). It seems possible that the aggregation idea from the study of soluble proteins might apply to some membrane proteins. Given the poor resolution at the junction of protein pairs, especially when the pairs are not ordered with respect to each other, we have no chemical detail on these contacts in Kv2.1. However, it seems likely that they are mediated by peptide–protein or peptide–peptide interactions.

### Slo1 Channels Interact Through Their Cytoplasmic Domains.

The high-conductance Ca^2+^-activated, voltage-dependent K^+^ channel Slo1 is a homotetramer like HCN1 and Kv2.1. Slo1 plays crucial roles in muscle contraction, neurotransmitter release, and hormone secretion (31). Slo1 channels have been shown to form large clusters both endogenously in neurons and under heterologous expression in kidney cells (32). These domains are proximal to clusters of the L-type calcium channel Cav1.3, allowing for efficient coupling between the two channels (32). How might Slo1 channels self-interact in these domains?

**Fig. 5.**
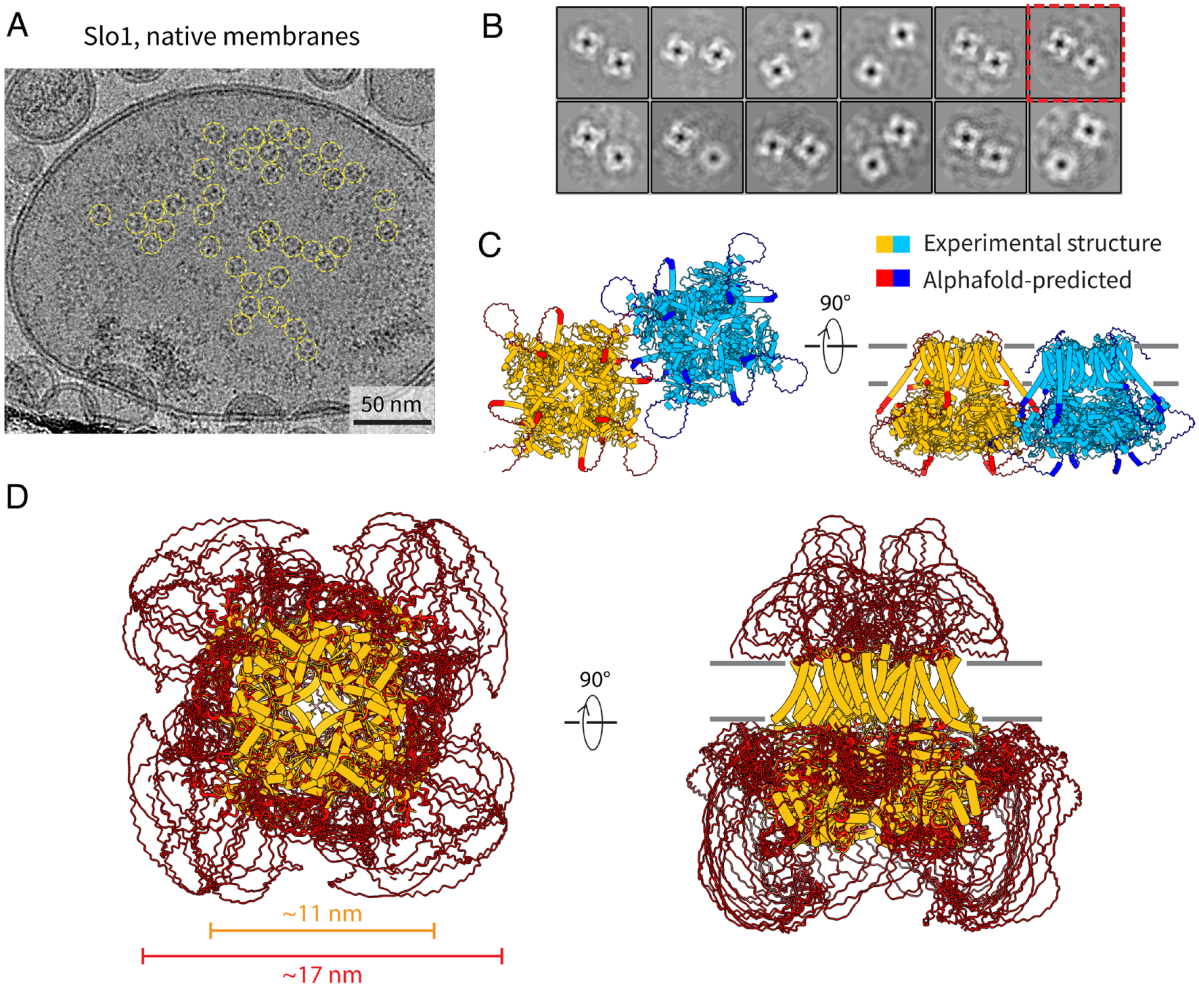
Clusters of Slo1 channels in NMVs. (*A*) Representative cryo-EM micrograph of NMVs containing Slo1 channels. (*B*) Two-dimensional class averages of proximal pairs of Slo1 channels. The class that was selected for panel *C* is marked by the red box. (*C*) Structural model of two interacting Slo1 channels shown in cartoon representation. The structure of Slo1 in membranes is shown in orange/light blue and the AlphaFold-predicted loops shown in red/dark blue. (*D*) Structural model of a single Slo1 tetramer showing the ordered transmembrane structure and cytoplasmic domain in orange, and the AlphaFold-predicted loops in red, to show the footprint of the channel. To emphasize the flexible nature of the loops, 10 AlphaFold-predicted models are overlaid.

We have previously determined the molecular structure of Slo1 in cell-derived NMVs (13). Here, we reanalyzed this dataset to study putative interactions between Slo1 channels. The channels clearly form clusters in NMVs (Fig. 5*A*). Two-dimensional class averages of tetramer pairs of Slo1 channels (Fig. 5*B*) show somewhat variable distances and orientations between channels, but in some classes, adjacent channels are close enough to interact through their cytoplasmic domains (Fig. 5*C*). In other classes, the channels are further apart, but could still interact through disordered loops in the cytoplasmic domain that are not visible in the 2D class averages (Fig. 5*D*). Thus, the case of Slo1 mirrors what we observed with Kv2.1—that contacts likely occur through peptide–peptide or peptide–protein interactions involving the cytoplasmic domain.

## Discussion

While it is well known that many membrane proteins at high concentrations form dense protein sheets (33–35), i.e., bulk phase clusters, we are motivated to study them now because we recently found that some membrane proteins form small self-assembled clusters in cell membranes at native concentrations of only a few proteins per square micrometer, i.e., HOTS (1). The formation of HOTS in cell membranes, where many other kinds of proteins are present, requires molecular specificity. Given that HOTS appear to enhance communication among components of a membrane signaling pathway, we hypothesize that they confer advantage and are genetically encoded (2). The proteins we analyze in the present study by reconstitution or in NMVs are known to form clusters in cell membranes (12, 17, 18, 22, 23, 32). The physical basis of their cluster formation has not been established, but self-assembly seems likely. We note that in addition to the single particle approach taken here, cryoelectron tomography (cryo-ET) is an emerging technique that is well poised to study such interactions in situ, as has been demonstrated for proteins such as ryanodine receptor (36), CatSper complexes (37), and GABAA receptors (38).

**Fig. 6.**
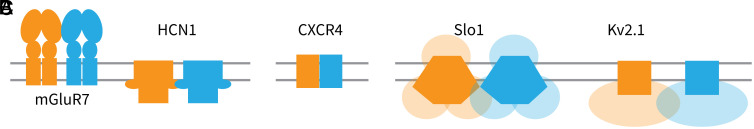
Schematic showing the different ways membrane proteins can self-interact. Membrane proteins contact each other in self-interacting clusters in a variety of ways, including through (*A*) ordered domains outside the membrane bilayer as in the cases of mGluR7 and HCN, (*B*) ordered TMDs (e.g., CXCR4), and (*C*) disordered domains outside the membrane bilayer (e.g., Slo1 and Kv2.1).

The first conclusion we reach is that different proteins make pairwise contacts in different ways (Fig. 6). In some cases (Fig. 6*A*) we observe contacts formed between structured domains on the extracellular side of the membrane bilayer (mGluR) or the intracellular side (HCN). None of the four proteins in this study formed extensive contacts through transmembrane helices inside the membrane, however, data from studies of others show that some class A GPCRs contact each other this way (Fig. 6*B*). For example, mutagenesis studies with some GPCRs point to transmembrane helix contacts (39, 40). Structures of the CXCR4 receptor and rhodopsin dimers showed that oligomers are formed mainly through transmembrane helices, although these two GPCRs differ in which helices contact each other to form the multimer (41, 42). Another kind of contact (Fig. 6*C*) we deduce from inspecting the clusters of Kv2.1 and Slo1 are those apparently mediated by IDRs. We cannot discern whether the IDRs bind to structured regions of a neighboring protein or to complementary sticky sequences contained within IDRs. Interactions of the latter type are thought to be important in the function of many soluble proteins, for example, to bring correct transcriptional proteins together in a transcriptionally active region (43, 44). We think it is possible that IDRs could serve a similar purpose to create active (high receptor concentration) regions where membrane signaling occurs (2).

This study does not characterize the specificity of interactions, but a central conclusion of our recent papers is that many membrane proteins recognize self (1, 2, 23, 24). What are the atomic origins of this specificity? To entertain this question, it is worth considering the following thought experiment. n monomers will oligomerize spontaneously if the free energy of n monomers exceeds the free energy of an nmer, and equilibrium will be reached when these free energies are equal. A free energy difference favoring oligomerization can be driven by cohesive interactions (i.e., sticky regions) formed at protein–protein interfaces when the proteins assemble to form an oligomer. Alternatively, interactions between a protein and its surrounding solvent molecules can drive oligomerization through, among other effects, an increase in entropy as ordered solvent molecules (water for extramembrane domains and lipid for intramembrane domains) are excluded from buried protein surfaces when oligomerization occurs. In either scenario, features of a protein’s sequence and/or structure would seem to underlie specificity, that is, its greater tendency to form homo-oligomers instead of hetero-oligomers through what we have called i–i interactions (2). It is likely that oligomerization is driven by both enthalpic and entropic contributions to the free energy difference between monomers and nmers, but in either case, specificity must map to features of the protein sequence and structure.

The binding of specific lipid molecules has been implicated in membrane protein oligomerization (41). Lipid involvement would not render oligomerization independent of protein structure and chemistry because ultimately lipid molecules interact with the protein through its physical properties. The idea that membrane protein oligomerization can also be mediated through mechanical properties of a bilayer is supported by simulations (45) and experiments pointing to the importance of membrane curvature (46) and hydrophobic mismatch (47). Such interactions also would have their origins in the properties of the lipid bilayer and the protein, for example, its shape, and thus map back to the protein. While it is likely that interactions mediated through mechanical properties of the bilayer play a role in membrane oligomerization, we imagine that the kind of specificity that allows two different class A GPCRs that have very similar structures to recognize “self” and not “other” is likely mediated by direct chemical interactions.

Whether the properties of a protein that encode self-recognition to form HOTS and bulk phase clusters are detectable at the level of protein sequence remains to be seen (48). If detectable, it might be possible to piece together interacting pairs of membrane proteins using sequence data to define signal pathways in membranes. Single dominant interactions like the one underlying the ordered clusters in HCN ought to be detectable at a sequence level. Likewise, if sticky complementary sequences underlie IDR interactions, they might be detectable. The multiple surfaces on mGluR ligand binding domains intuitively seem more challenging because we imagine that scattered residues that could mediate cohesion—for example, a distributed array of charged residues—might be enough to make them stick, but since this could occur in so many ways this kind of contact site might be difficult to detect at the sequence level. We raise these ideas as a challenge to those studying covarying sets of amino acids.

It is well known that many membrane proteins aggregate into sheets (bulk phase) at high concentrations. In fact, the phenomenon is so familiar to us that we might tend to ignore it as biologically unimportant. But HOTS in the presence of numerous competing proteins implies specificity in some of these interactions, leading us to inspect membrane protein self-interactions carefully. Some interactions may turn out to be serendipitous, but others will likely play a biological role. Covalently bonded biomolecules are cubic nanometer sized, whereas the smallest living cells are a billion times that volume, entirely built up through noncovalent assemblies of the much smaller covalent structures. In other words, much of life’s chemistry is noncovalent. We expect there ought to be rules governing the spontaneous assembly of proteins evolved to function in groups. Analyzing noncovalent assemblies of membrane proteins benefits from the simplification provided by a two-dimensional environment.

## Materials and Methods

### Cell Lines.

Sf9 (*Spodoptera frugiperda* Sf21) cells were used for production of baculovirus and were cultured in Sf-900 II SFM medium (GIBCO) supplemented with 100 U/mL penicillin and 100 U/mL streptomycin at 27 °C under atmospheric CO_2_.

HEK293S GnTl^−^ cells were used for protein expression and were cultured in Freestyle 293 medium (GIBCO) supplemented with 2% fetal bovine serum, 100 U/mL penicillin, and 100 U/mL streptomycin at 37 °C in 8% CO_2_.

### Expression and Purification of mGluR7.

The full-length human GRM7 gene (Addgene plasmid #66392) was cloned into the pEG BacMam vector for expression in HEK293S GnTI^−^ cells. The endogenous signal peptide at the N terminus was replaced with a cleavable hemagglutinin signal peptide, followed by an Alfa-tag for purification. HEK293S GnTI^−^ cells were infected with 10% (v/v) recombinant BacMam virus at a density of ~3 × 10^6^ cells/mL. After 15 h, 10 mM sodium butyrate was added to induce expression at 30 °C. Cells were harvested 48 h postinduction by centrifugation at 1,000× g for 10 min at 4 °C. The purification method is modified from previous publications with slight changes (11, 49). Cell pellets from 1 L culture were resuspended in 50 mL lysis buffer (50 mM Tris-HCl pH 7.5, 150 mM NaCl, 20 μg/mL leupeptin, 20 μg/mL pepstatin A, 2 mM benzamidine, 4 μg/mL aprotinin, 20 μg/mL E-64, 1 mg/ml AEBSF, and ~200 μg/mL DNase. The suspension was homogenized using a Dounce homogenizer and subsequently clarified by centrifugation at 39,000× g for 1 h at 4 °C.

The crude membrane fraction was resuspended in 50 mL membrane resuspension buffer using a Dounce homogenizer, followed by solubilization with an equal volume of solubilization buffer [50 mM Tris-HCl pH 7.5, 300 mM NaCl, 1% (w/v) n-dodecyl-β-D-maltopyranoside (DDM) (Anatrace):0.2% (w/v) cholesteryl hemisuccinate (CHS) (Sigma-Aldrich)]. The solubilization reaction was carried out at 4 °C for 3 h with gentle agitation. The solubilized membranes were then clarified by centrifugation at 39,000× g for 30 min at 4 °C. The clarified supernatant was incubated with 1 mL Alfa-nanobody resin at 4 °C for 2 h under gentle rotation. The resin was washed with 20 mL wash buffer [50 mM Tris-HCl pH 7.5, 500 mM NaCl, 0.05% (w/v) DDM:0.01% (w/v) CHS]. Bound protein was eluted using 10 mL elution buffer [50 mM Tris-HCl pH 7.5, 150 mM NaCl, 0.05% (w/v) DDM:0.01% (w/v) CHS, 1 mg/mL Alfa-peptide]. The eluate was concentrated to ~500 μL using a 100-kDa molecular weight cutoff centrifugal filter (Amicon, Millipore) and loaded onto a Superose 6 Increase 10/300 GL column (Cytiva) pre-equilibrated in size-exclusionchromatography (SEC) buffer [50 mM Tris-HCl pH 7.5, 150 mM NaCl, and 0.05% (w/v) DDM:0.01% (w/v) CHS]. Fractions containing mGluR7 were pooled, concentrated, and used for reconstitution.

### mGluR7 NMV Preparation.

The preparation is modified from previously published methods (13, 50). One liter of cultured HEK293S GnTI^−^ cells at a concentration of approximately 3 to 4 × 10^6^ cells/mL was collected by centrifugation at 3,000× g for 10 min. The resulting cell pellet was resuspended in 200 mL of GPMV buffer containing 10 mM K-HEPES (pH 7.4), 140 mM NaCl, 10 mM KCl, and 2 mM CaCl_2_. After a second centrifugation under the same conditions, the pellet was resuspended in 400 mL of the same buffer, supplemented with 7.5 mM NEM (Thermo Scientific™ Pierce™, 23030). The suspension was distributed into four 250 mL baffled flasks, with 100 mL per flask, and incubated at 37 °C while shaking at 130 rpm for 2 h. After incubation, the flasks were briefly shaken manually (~30 s) to further aid vesicle detachment. To remove intact cells and larger GPMVs, the suspension was centrifuged at 3,000× g for 10 min at 4 °C. The supernatant was supplemented with 10% glycerol to prevent vesicle aggregation. This mixture was sonicated using a probe sonicator (Branson, 1/2” tip, Branson102-C converter) set to 40% amplitude for three 30-s bursts, allowing ~1-min cooling intervals on ice between pulses.

Vesicles were then pelleted by ultracentrifugation at 100,000× g for 40 min at 4 °C using a Ti70 rotor. Each pellet, obtained from ~25 mL of sample, was gently resuspended in ~1 mL of GPMV buffer containing 10% glycerol by directing the buffer toward the pellet using a 200 μL pipette tip. The suspension was then sonicated in a Branson M1800 water bath sonicator using short pulses (~10 s each) at room temperature, until the solution became opalescent (typically within 30 to 60 s). To eliminate aggregates, the suspension was centrifuged at 3,500× g for 10 min at 4 °C. The clarified supernatant (~20 mL) was then incubated overnight at 4 °C with 1 mL of ALFA Selector CE resin (NanoTag), pre-equilibrated in GPMV buffer containing 10% glycerol. The following day, the resin was batch-washed twice with ~20 mL of the same buffer, pelleted at 1,000× g for 1 min at 4 °C, and then washed again with 15 mL of glycerol-supplemented buffer. The resin was transferred to a gravity column, washed with an additional 5 mL of buffer containing glycerol, and then rinsed with 15 mL of buffer lacking glycerol. Elution was performed in three steps using GPMV buffer containing 0.2 mM ALFA peptide (NanoTag): first with 5 column volumes (CVs), followed by another 5 CVs, and then 3 CVs. Each elution step involved a 30-min incubation at room temperature. Eluted fractions were kept on ice and pooled. The combined sample was concentrated using an Amicon 2 mL centrifugal filter unit (100 kDa MWCO) to reach an approximate absorbance of OD_280_ ~2.

### Expression and Purification of HCN1.

Human HCN1 with a C-terminal truncation (residues 636 to 865) was expressed and purified as described before (19) with slight modifications. Briefly, HCN1 was expressed using the BacMan system in HEK293S GnTI^−^ cells. After lysing the cells and collecting the membrane fraction, protein was solubilized in buffer containing 1.5%:0.3% lauryl maltose neopentyl glycol (LMNG):CHS (wt/vol). HCN1 was affinity purified on a green fluorescent protein (GFP)-nanobody resin; then, the GFP tag was cleaved off using preScission protease, and the eluate was collected and concentrated at 3,000× g and 4 °C (100 kDa cutoff spin filter) for SEC. The final SEC purification step used a Superose 6 Increase column (10/300 GL) pre-equilibrated with SEC buffer (10 mM Tris pH 8.0, 150 mM KCl, 0.01%:0.002% LMNG:CHS, and 5 mM DTT). Fractions containing HCN1 were pooled and concentrated at 2,000× g and 4 °C to an A_280_ of ~2 mg/mL.

### Expression and Purification of K_v_2.1.

Full-length human K_v_2.1 with a C-terminal GFP-His_6_ tag linked by a preScission protease (PPX) site was expressed as detailed before, using the BacMan system in HEK293S GnTI^−^ cells (51). The final SEC purification step used a Superose 6 Increase column (10/300 GL) pre-equilibrated with SEC buffer (10 mM Tris pH 8.0, 150 mM KCl, 0.03%:0.006% DDM:CHS, and 5 mM DTT). Fractions containing K_v_2.1 were pooled and concentrated at 2,000× g and 4 °C to an A_280_ of ~2 mg/mL.

### Slo1 NMVs.

No new samples were prepared for studying Slo1 in NMVs—we simply reanalyzed existing cryo-EM data. The purification protocol for has been reported before (13).

### Reconstitution of mGluR7, HCN1, and K_v_2.1 into Synthetic Liposomes.

Purified mGluR7 protein in a buffer containing 50 mM Tris-HCl (pH 7.5), 150 mM NaCl, 0.05% (w/v) DDM, and 0.01% (w/v) CHS, was mixed with small unilamellar vesicles (SUVs) prepared from POPC (Avanti Polar Lipids) at 10 mg/mL. The liposomes were suspended in reconstitution buffer (50 mM Tris-HCl, pH 7.5, and 150 mM NaCl) and permeabilized by adding DDM to a final concentration of 0.5% (w/v). Protein and lipids were mixed at a mass ratio of 1:50 (protein:lipid), and detergent removal was carried out by dialysis using a 50 kDa molecular weight cutoff membrane. Dialysis was performed over the course of 5 d at 4 °C, with two complete buffer exchanges per day to gradually remove the detergent and promote incorporation of the protein into the lipid bilayer.

Preparation of liposomes containing HCN1 or K_v_2.1 was carried out largely as described before (51). In brief, purified proteins were reconstituted into liposomes consisting of POPC (Avanti Polar Lipids) (51, 52). SUVs were produced by bath sonication in reconstitution buffer (10 mM Tris pH 8.0 and 150 mM KCl) and a low concentration (0.2% wt/wt for 10 mg/mL lipids) of the detergent C_12_E_10_ (in the case of Kv2.1) or LMNG (in the case of HCN1) was added to the liposome suspension. Purified K_v_2.1 or HCN1 was mixed with lipids at a protein:lipid ratio of 1:20 (wt/wt) and detergent was removed over the course of ~20 h using three rounds of Bio-beads. The final concentration of lipids in the sample was 5 mg/mL.

### Preparation of Cryo-EM Grids for Vesicle Samples.

To prepare cryo-EM grids, the proteoliposome solution was applied onto a glow-discharged Quantifoil R1.2/1.3 400 mesh holey carbon Au grid. After incubating the sample on the grid for 3 min at 20 °C with a humidity of 100%, the grid was manually blotted from the edge of the grid using a filter paper. Another 3.5 µL of the vesicle solution was applied to the same grid for 20 s (53), and then, the grid was blotted for 3 s with a blotting force of 0 and flash frozen in liquid ethane using a FEI Vitrobot Mark IV (FEI).

### Cryo-EM Data Collection and Processing for mGluR7 in NMVs.

Cryo-EM data for mGluR7 in NMVs were acquired using a 300-keV FEI Titan Krios transmission electron microscope equipped with a Selectris X energy filter (6 eV slit width) and a Falcon 4i direct electron detector operated in electron event representation (EER) mode. Data were collected in SerialEM (54) using a 3 × 3 multishot pattern with three shots per hole. Images were recorded at a nominal magnification of 105,000×, corresponding to a physical pixel size of 1.196 Å. The defocus range was set between –1.5 and –2.0 µm, and the total electron dose was ~42 electrons/Å^2^, distributed over 2,043 EER frames. A total of 11,524 movies were collected.

All movies were gain-normalized and motion-corrected using cryoSPARC’s Patch Motion Correction tool (55). Contrast transfer function (CTF) parameters were estimated using cryoSPARC’s Patch CTF Estimation tool. All subsequent processing steps were performed using dose-weighted micrographs.

Initial particle picking was performed using a combination of manual picking and Topaz (56), resulting in 1,164,582 particles. These particles were subjected to multiple rounds of deep 2D classification to remove junk and low-quality classes. A total of 930,353 particles were selected for ab initio reconstruction into three classes. The best-resolved class, containing 258,446 particles, was chosen for further processing.

Final refinement was performed using nonuniform refinement followed by local refinement in cryoSPARC without imposing symmetry (C1). The final map, based on 253,946 particles, reached a global resolution of 4.1 Å as determined by the gold-standard Fourier shell correlation (FSC) at the 0.143 criterion.

### Cryo-EM Data Collection and Processing for mGluR7 in Reconstituted Vesicles.

Cryo-EM data for mGluR7 reconstituted into liposomes were acquired using a 300-keV FEI Titan Krios transmission electron microscope equipped with a Selectris X energy filter (6 eV slit width) and a Falcon 4i direct electron detector. Data collection was performed in SerialEM (54) using a 3 × 3 multishot pattern with four shots per hole. Images were recorded at a nominal magnification of 165,000×, corresponding to a physical pixel size of 0.743 Å. The defocus range was set between –1.0 and –2.0 µm, and the total electron dose was ~60 electrons/Å^2^. Exposure times were 3.355 s for one grid and 3.666 s for a second grid, yielding 6,736 and 16,862 movies, respectively, for a total of 23,598 movies.

All movies were gain-normalized and motion-corrected using cryoSPARC’s Patch Motion Correction tool (55). CTF parameters were estimated using cryoSPARC’s Patch CTF Estimation tool. All subsequent steps were performed using dose-weighted micrographs.

Initial particle picking was performed using the Blob picker in cryoSPARC. Selected 2D classes showing clear protein features were used to train a Topaz neural network model (56), which was then applied to repick particles across the full dataset. After removal of duplicates and low-quality particles, multiple rounds of 2D classification were performed to enrich for homogeneous classes.

A total of 804,110 particles were selected and subjected to nonuniform refinement in cryoSPARC. The resulting volume was further processed using three-dimensional (3D) classification without alignment to identify the best-resolved class. Final refinement of 498,211 particles was conducted using nonuniform refinement and local refinement in cryoSPARC with no imposed symmetry (C1). The final map reached a global resolution of 4.3 Å, based on the gold-standard FSC at the 0.143 criterion.

### Cryo-EM Data Acquisition and Processing for HCN1 and Kv2.1.

Data for the HCN1 liposomes were collected on a 300-keV FEI Titan Krios 3 microscope located at the HHMI Janelia Research Campus. The microscope was equipped with a cold field emission gun, a Thermo Scientific Selectris X energy filter, and a Thermo Scientific Falcon 4i camera. A total of 18,684 movies were recorded on a single Quantifoil grid using SerialEM (54), but only a third of the movies (6,228) were ultimately processed. The movies were recorded with a physical pixel size of 0.94 Å and a target defocus range of −1.0 to −2.0 µm. The total exposure time was ~2 s (fractionated following the EER format) with a cumulative dose of ~60 e^−^/Å^2^.

Data for the K_v_2.1 liposomes were collected on a 300-keV FEI Titan Krios 2 microscope located at the HHMI Janelia Research Campus. The microscope was equipped with a GIF BioQuantum energy filter and a Gatan K3 camera. A total of 18,843 movies were recorded on a single Quantifoil grid in superresolution mode using SerialEM. The movies were recorded with a physical pixel size of 0.831 Å (superresolution pixel size of 0.4155 Å) and a target defocus range of −1.0 to −2.0 µm. The total exposure time was ~2 s (fractionated into 50 frames) with a cumulative dose of ~60 e^−^/Å^2^.

The data processing workflow for HCN1 and Kv2.1 used cryoSPARC v4 (55) as described before (51, 52). The movies were gain-normalized and corrected for full-frame and sample motion using the Patch motion correction tool. Contrast transfer function parameters were estimated from the motion-corrected micrographs using the Patch CTF estimation tool. Particle picking was initially carried out using the Blob picker. 2D classes with clear protein density were used to train a TOPAZ picking model (56), which was then used to pick additional particles. Particles with clear protein density after 2D classification were pooled and duplicate picks were removed. An ab initio model was generated from 2D classes with clear secondary structure features and 3D classification and refinement was carried out in cryoSPARC.

The final C4-symmetric cryo-EM density map for HCN1 used 270,241 particles and had a nominal global resolution of 3.7 Å (FSC = 0.143). The final C4-symmetric cryo-EM density map for Kv2.1 used 746,888 particles and had a nominal global resolution of 3.6 Å (FSC = 0.143).

### Model Building and Refinement.

All structural models in this work used existing structures of isolated mGluR7 (11), HCN1 (19), Kv2.1 (25), or Slo1 (13) as a starting point. In the case of mGluR7, the map was of sufficiently high quality to refine the structure. For HCN1 and Kv2.1, the previously reported structures were fit into the map as a rigid body. Finally, there was no need to recalculate a new map for Slo1 as the reported map was derived from the same dataset.

## Supplementary Material

Appendix 01 (PDF)

## Data Availability

The cryo-EM density map of mGluR7 in native membrane vesicles has been deposited in the electron microscopy data bank under accession code EMD-70614 and the corresponding model has been deposited in the protein data bank under accession code 9OMO. The cryo-EM density map of mGluR7 in reconstituted vesicles has been deposited in the electron microscopy data bank under accession code EMD-70615 and the corresponding model has been deposited in the protein data bank under accession code 9OMP. The cryo-EM density maps of HCN1 and Kv2.1 in reconstituted vesicles have been deposited in the electron microscopy data bank under accession codes EMD-70635 and EMD-70634, respectively. The cryo-EM density map and corresponding atomic model for Slo1 have been previously reported (13).
