# Supplementary material for: Molecular contacts in self-assembling clusters of membrane proteins
Source: Proc Natl Acad Sci U S A. 2025 Jun 23;122(26):e2507112122. doi: 10.1073/pnas.2507112122 (PMC12232663; doi:10.1073/pnas.2507112122)
Supplement: Supplementary file 1 — Appendix 01 (PDF) [file pnas.2507112122.sapp.pdf]

Supporting Information for

Molecular contacts in self-assembling clusters of membrane proteins.

Venkata Shiva Mandala[1,a], Ziao Fu[1,a, b] and Roderick MacKinnon[1, *].

[1]Laboratory of Molecular Neurobiology and Biophysics, Howard Hughes Medical Institute, The Rockefeller University, New York, NY, United States.

[a]V.S.M. and Z.F. contributed equally to this work.

[b]Current address: Department of Cell Biology and Physiology, Washington University School of Medicine, St. Louis, MO, United States.

*Correspondence to: Roderick MacKinnon.

Email: mackinn@rockefeller.edu.

This PDF file includes:

Figures S1 to S2

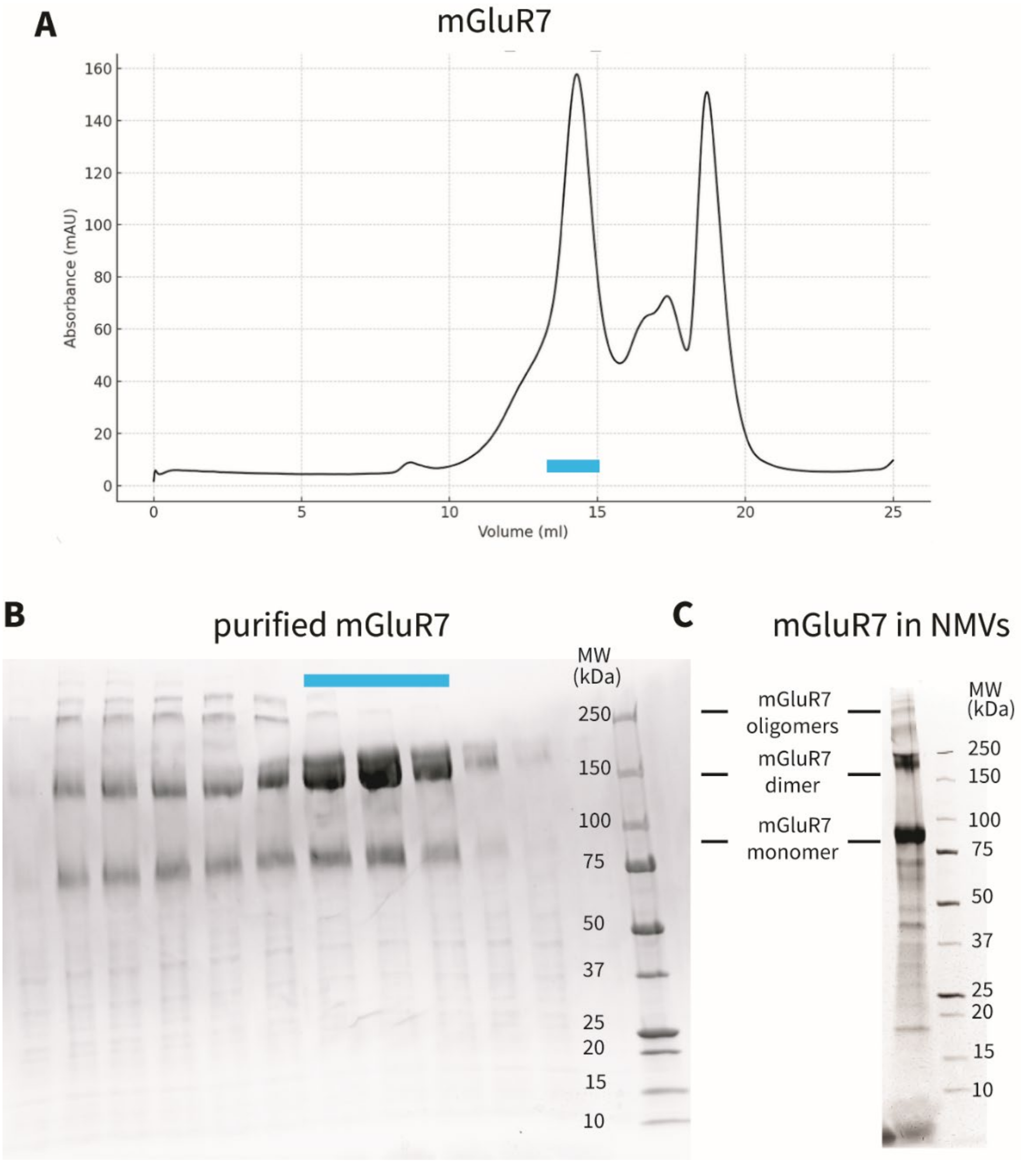

Figure S1. Purification and characterization of mGluR7 for cryo-EM studies.

(**A**) Size-exclusion chromatogram (SEC) of detergent-solubilized mGluR7 on a Superose 6 Increase column. The fractions highlighted in blue (14.5–16.5 mL) were pooled for reconstitution into liposomes. (**B**) SDS-PAGE analysis of the SEC fractions shows purified mGluR7, which was used for reconstitution into synthetic vesicles. Selected fractions are indicated in blue. (**C**) SDS-PAGE of mGluR7 isolated in native membrane vesicles, showing mGluR7 is the dominant protein in the preparation.

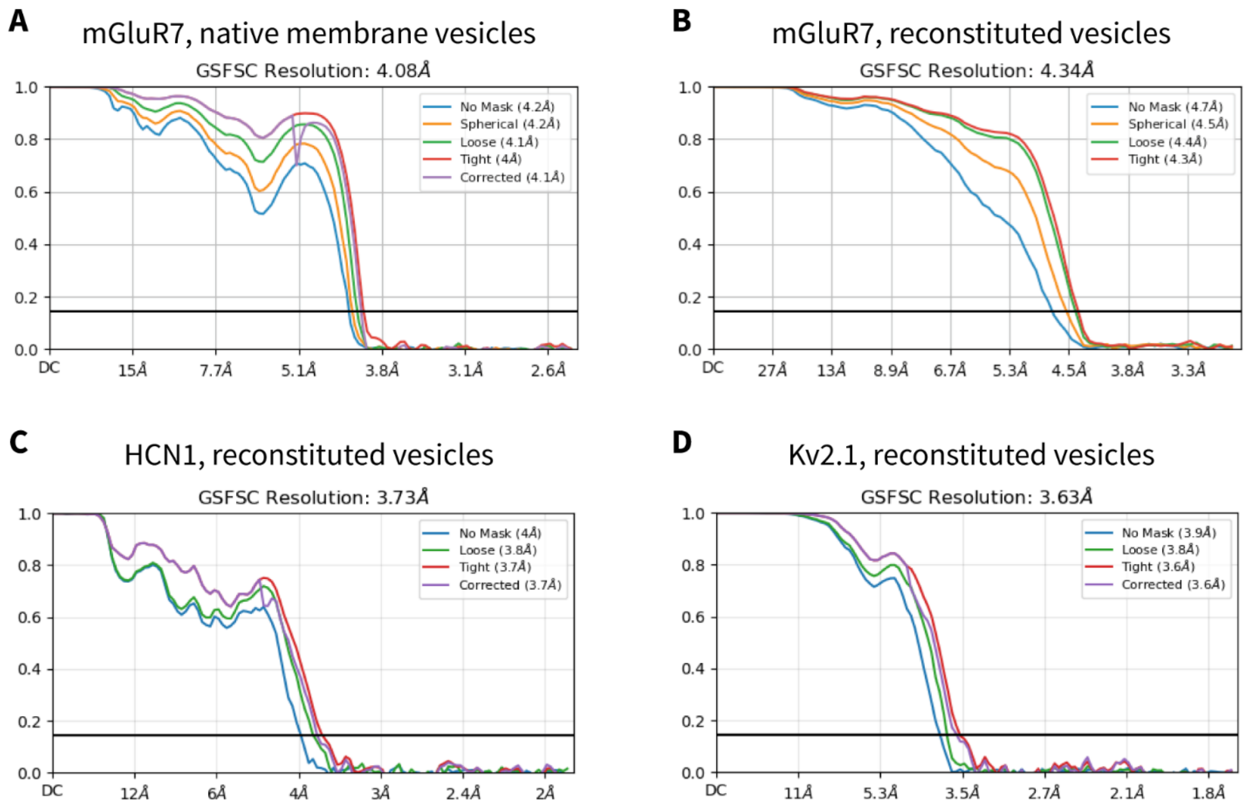

Figure S2. Fourier shell correlation curves for cryo-EM density maps.

CryoSPARC-derived Fourier Shell Correlation (FSC) curves for (**A**) mGluR7 in native membrane vesicles, (**B**) mGluR7 in reconstituted vesicles, (**C**) HCN1 in reconstituted vesicles, and (**D**) Kv2.1 in reconstituted vesicles. The nominal resolution at the gold-standard criterion (FSC=0.143, black line) is indicated for each plot.
